# Improvement of transpiration estimation based on a two-leaf conductance-photosynthesis model with seasonal parameters for temperate deciduous forests

**DOI:** 10.3389/fpls.2023.1164078

**Published:** 2023-05-08

**Authors:** Jiaxin Jin, Ying Liu, Weiye Hou, Yulong Cai, Fengyan Zhang, Ying Wang, Xiuqin Fang, Lingxiao Huang, Bin Yong, Liliang Ren

**Affiliations:** ^1^ College of Hydrology and Water Resources, Hohai University, Nanjing, China; ^2^ Key Laboratory of Water Big Data Technology of Ministry of Water Resources, Hohai University, Nanjing, China; ^3^ National Earth System Science Data Center, National Science & Technology Infrastructure of China, Beijing, China; ^4^ Tourism and Social Administration College, NanJing XiaoZhuang University, Nanjing, China; ^5^ State Key Laboratory of Resources and Environment Information System, Institute of Geographic Sciences and Natural Resources Research, Chinese Academy of Sciences, Beijing, China

**Keywords:** two-leaf scheme, ball-berry model, stomatal conductance, transpiration, light use efficiency, seasonal variability, temperate deciduous forests

## Abstract

**Introduction:**

Conductance-photosynthesis (G_s_-A) models, accompanying with light use efficiency (LUE) models for calculating carbon assimilation, are widely used for estimating canopy stomatal conductance (G_s_) and transpiration (T_c_) under the two-leaf (TL) scheme. However, the key parameters of photosynthetic rate sensitivity (g_su_ and g_sh_) and maximum LUE (ϵ_msu_ and ϵ_msh_) are typically set to temporally constant values for sunlit and shaded leaves, respectively. This may result in T_c_ estimation errors, as it contradicts field observations.

**Methods:**

In this study, the measured flux data from three temperate deciduous broadleaved forests (DBF) FLUXNET sites were adopted, and the key parameters of LUE and Ball-Berry models for sunlit and shaded leaves were calibrated within the entire growing season and each season, respectively. Then, the estimations of gross primary production (GPP) and T_c_ were compared between the two schemes of parameterization: (1) entire growing season-based fixed parameters (EGS) and (2) season-specific dynamic parameters (SEA).

**Results:**

Our results show a cyclical variability of ϵ_msu_ across the sites, with the highest value during the summer and the lowest during the spring. A similar pattern was found for g_su_ and g_sh_, which showed a decrease in summer and a slight increase in both spring and autumn. Furthermore, the SEA model (i.e., the dynamic parameterization) better simulated GPP, with a reduction in root mean square error (RMSE) of about 8.0 ± 1.1% and an improvement in correlation coefficient (r) of 3.7 ± 1.5%, relative to the EGS model. Meanwhile, the SEA scheme reduced T_c_ simulation errors in terms of RMSE by 3.7 ± 4.4%.

**Discussion:**

These findings provide a greater understanding of the seasonality of plant functional traits, and help to improve simulations of seasonal carbon and water fluxes in temperate forests.

## 1 Introduction

Transpiration (T_c_) accounts for a major fraction, about 80%, of land surface evapotranspiration, playing a crucial role in the water cycle (Jasechko et al., 2013). Accurately determining T_c_ is critical for comprehending the regional water and energy budget as well as for understanding ecological processes (Shen et al., 2015; Li et al., 2018; Ding et al., 2022). Although direct observation methods, such as stem flow meters and infiltrators, can provide relatively reliable data, their scope of observation is limited, only allowing for T_c_ estimation at a single plant or field scales, making it difficult to estimate T_c_ at regional and global scales (Wang and Dickinson, 2012). With the advancement of remote sensing technology, indirect estimation of T_c_ has drawn extensive attention (Chen and Liu, 2020). By integrating satellite observations with mechanistic models, T_c_ can be detected at a larger scale, providing a valuable insight of hydrological processes (Liu et al., 2022).

The Penman-Monteith (P-M) equation, which integrates available energy, surface resistances and environmental variables (e.g., dryness of the atmosphere), is widely used to estimate T_c_ (Monteith and Unsworth, 2013). Canopy stomatal conductance (G_s_, the inverse of resistance) is particularly critical in the calculation of T_c_ because of its sensitivity to environmental (e.g., air temperature, relative humidity, CO_2_ concentration, etc.) and phenological (e.g., leaf ontogeny and canopy development) variables (Zhang K. et al., 2019; Jin et al., 2022). Hence, how to determine G_s_ is an important prerequisite for accurate estimation of T_c_. Stomata are the main channel of transpiration, controlling the water vapor flow from soil to atmosphere through vegetation, as well as the rate of carbon dioxide (CO_2_) entering leaf flesh tissue from the atmosphere (Hetherington and Woodward, 2003). Leaf stomatal conductance (g_s_) is regulated by a combination of abiotic and biotic factors, and G_s_ can be recognized as the sum of all leaf conductance in the entire canopy (Luo et al., 2018). That is, both g_s_ calculation and the scaling transformation from leaf to canopy level are necessary to obtain G_s_.

Ball et al. (1987) related stomatal conductance and leaf photosynthesis rate through the linear relationship, and proposed the Ball-Barry (B-B) model, which considers the effects of atmospheric humidity and CO_2_ concentration on stomatal conductance, to estimate g_s_. This conductance-photosynthesis (g_s_-A) model requires fewer empirical parameters, and is widely used at diverse scales (Miner et al., 2017), accompanying with photosynthesis models for calculating carbon assimilation (Ryu et al., 2011; Zhang Y. et al., 2019), e.g., light use efficiency (LUE) models (Pei et al., 2022). For scaling from leaf to canopy, the typical approach was the big-leaf (BL) scheme, in which the canopy is assumed to be a large leaf and g_s_ is multiplied by leaf area index (LAI) (Kimball et al., 1997). However, the real canopy has more than one layer of leaves, and the leaves shade each other. Sellers et al. (1992) took into account the variation of light in the canopy and then improved the big-leaf scheme with an exponential function of LAI to calculate the G_s_. However, the g_s_ of the top leaves is limited by the carboxylation capacity, and that of the bottom leaves is mainly limited by radiation. This improvement cannot express the actual condition of canopy. The two-big-leaf (TBL) scheme was proposed to improve this issue by dividing the canopy into two large leaves, sunlit and shaded leaves, and requires an artificial upscaling of leaf-scale physiological parameters to their counterparts to obtain G_s_ for each leaf group, respectively (Wang and Leuning, 1998; Dai et al., 2004). The TBL scheme reflects the difference in radiation between sunlit and shaded leaves in the canopy and is more realistic than the BL scheme. However, the TBL model scales g_s_ to G_s_ through LAI with certain incompatibilities, and Luo et al. (2018) showed that G_s_ and the product of g_s_ and LAI were not equivalent, and thereby this up-scaling approach also brings uncertainty. Chen et al. (1999) proposed the two-leaf (TL) scheme, in which physiological parameters are calculated for a representative sunlit leaf and a representative shaded leave, respectively, and multiplied by the corresponding LAI values to upscale to canopy-scale counterparts for estimating G_s_. The TL model uses leaf-scale stomatal conductance and photosynthetic capacity (e.g., LUE) to ensure the consistency of the parameters inherent in the g_s_-A model.

Model theory and parametrization together determine modeling accuracy. The current theoretical development of the g_s_-A models has become more prominent (Leuning et al., 1995; Katul et al., 2010; Medlyn et al., 2011). However, the parametrized schemes remain less robust. Generally, the key parameters in the g_s_-A models are temporal constant (Miner et al., 2017), especially the model with the two-leaf scheme (Lawrence et al., 2011; Li et al., 2019). Previous studies have reported that hydraulic and photosynthetic parameters varied with time, showing a higher value in mature leaves relative to young and old leaves (Wilson et al., 2001; Albert et al., 2018; Chavana-Bryant et al., 2019). Therefore, the fixed parametrization may hamper the ability to accurately estimate T_c_.

Considering the seasonality of the physiological variables, this study aimed to investigate performances of the TL-based g_s_-A models with different parametrization schemes (i.e., the fixed vs. dynamic parametrization schemes). Towards this aim, the TL-LUE (He et al., 2013) and TL B-B model (Li et al., 2019) were adopted, as well as flux- and satellite-based observations at three temperate deciduous broadleaf forests (DBF) FLUXNET sites. Two specific questions are answered in this study: (1) Do the key parameters of photosynthesis and stomatal conductance modeling vary with season? (2) Could the seasonal dynamic parametrized scheme effectively improve the estimations of GPP, G_s_ and thereby T_c_.

## 2 Materials and methods

### 2.1 Flux data

**Figure 1:**
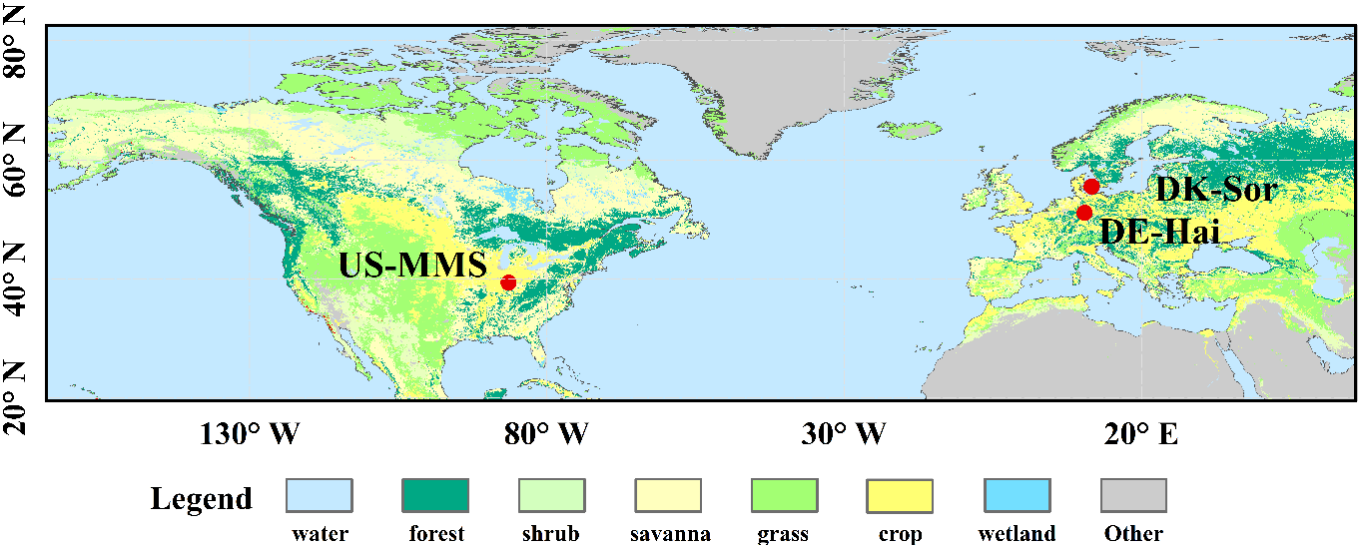
Spatial distribution of the study sites. Details of the sites are described in **Table 1**.

Three typical DBF sites from the FLUXNET2015 dataset (i.e., DE-Hai, DK-Sor and US-MMS) were used in this study, and the spatial distribution and details of the sites are shown in **Figure 1** and **Table 1**, respectively. DBF is a widespread vegetation type in the temperate zone, with significant seasonal variation in phenology. The site selection considered the length and quantity of the data records and vegetation growth conditions (Zhang et al., 2018). In this paper, half-hourly/hourly flux and climate data were obtained, including incoming/outgoing short/long-wave radiation, temperature, precipitation, wind speed, vapor pressure deficit (VPD), relative humidity, ground/latent/sensible heat fluxes, CO_2_, and GPP (“GPP_DT_VUT_REF”) derived from the net ecosystem exchange (NEE) (Reichstein et al., 2005). Half-hourly/hourly observations were subjected to strict quality control following that described in Jin et al. (2022), and only daytime observations with a shortwave incident radiation > 5 W/m² were used for analysis (Yebra et al., 2013). The resulting daytime data was then integrate into daily data. The method for calculating daily T_c_ was also consistent with that of Jin et al. (2022), in which the underlying water-use efficiency (uWUE) is used to identify the ratio of T_c_ to ET (Zhou et al., 2015, 2016a). More details of the theory and the applications are described in Zhou et al. (2015; 2016a) and Jin et al. (2022).

**Table 1:** Summary of the three deciduous broadleaf forest (DBF) FLUXNET sites.

Site ID	LAT	LON	H	Z	Period of Record	Spring	Summer	Autumn	Refs.
**DE-Hai**	51.08	10.45	23	44	2003-2009	56-156	157-250	251-336	Knohl et al. (2003)
**DK-Sor**	55.49	11.64	25	43	2006-2013	62-163	164-255	256-341	Pilegaard et al. (2011)
**US-MMS**	39.32	-86.41	27	48	2002-2014	58-157	158-250	251-317	Schmid et al. (2000)

For each site, Latitude (LAT, °N), Longitude (LON, °E), heights of canopy (H, m) and measurement (Z, m), period of record, and start and end dates of the three seasons (day of year, doy).

### 2.2 Remote sensing data

The LAI and fraction of absorbed photosynthetically active radiation (FPAR) data used in this study were obtained from version 4 of the Global Land Surface Satellite (GLASS) products with a spatial resolution of 0.05° and a temporal resolution of 8 days (Liang et al., 2021). The LAI data were generated by applying generalized regression neural networks on multiple satellite LAI time series and MODIS surface reflectance data (Xiao et al., 2014). The FPAR were derived from the LAI using a table lookup method (Xiao et al., 2015). To ensure consistency with the observed data, the 8-day LAI and FPAR data were interpolated to daily-scale using linear interpolation and improved by Savitzky-Golay filtering (Chen et al., 2004).

### 2.3 Model description

This study used the two-leaf (TL) Ball-Berry model (B-B) (Li et al., 2019) to calculate G_s_, accompanying with the TL light use efficiency model (TL-LUE, He et al., 2013) for calculating GPP of sunlit and shaded leaves. After that, the P-M equation was utilized to estimate T_c_ (Mu et al., 2011).

**Figure 2:**
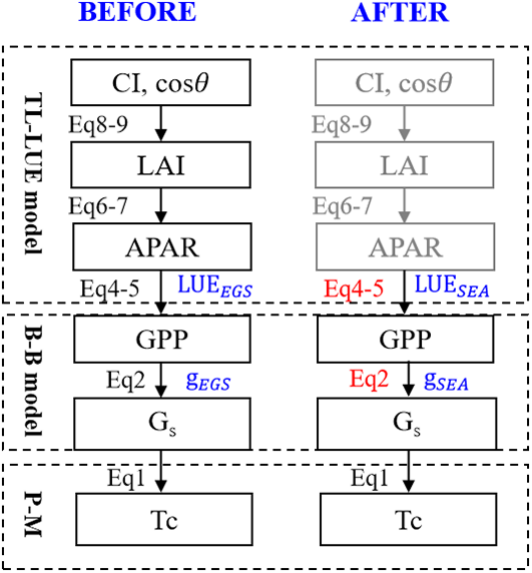
Improved algorithms for transpiration estimation based on the two-leaf scheme. EGS and SEA indicate the entire growth season-based fixed parameters and season-specific dynamic parameters, respectively.

The details of the technical flow are presented in **Figure 2**. LAI and absorbed photosynthetically active radiation (APAR) were calculated under the TL scheme for both sunlit and shaded leaves. The growing season was identified by multi-year temperature and were divided into three seasons (spring, summer and autumn) for each site (Park et al., 2018). We conduct 1000 randomized experiments for model calibration and validation. In each experiment, 70% of each seasonal data was selected as the training group, and the remaining data served as the testing group. The physiological parameters of the model were calibrated using two parameterized schemes: the entire growth season-based fixed parameterization (EGS) and season-specific dynamic parameterization (SEA). Estimated GPP, G_s_ and T_c_ with the two parameterization schemes were evaluated using the independent observations.

#### 2.3.1 P-M equation

The P-M equation introduces aerodynamic conductivity and surface conductivity to control the evaporation process and integrates biological and physical mechanisms (Monteith and Unsworth, 2013). It is formulated as Eq. (1) in this study:


(1) $$T_{c}=\frac{\left(s\cdot A_{c}+\rho \cdot C_{p}\cdot FPAR\cdot VPD\cdot G_{a}\right)\cdot \left(1-Fwet\right)}{s\cdot \gamma \cdot \left(1+\frac{G_{a}}{G_{s}}\right)}\cdot \frac{1}{\lambda }$$

where *s* is the slope of the saturation water vapor pressure vs. temperature curve ( ${\text{kP}}_{\text{a}}$ ·°C^-1^); *Ac* is the energy allocated to the canopy; ρ is the air density (kg·m^-3^); $C_{P}$ is the constant pressure specific heat (MJ·kg^-1^·°C^-1^); *Fwet* is the water coverage, which is zero for relative humidity (RH) less than 70% and RH^4^ for greater than 70% (Mu et al., 2011); γ is the wet and dry meter constant ( ${\text{kP}}_{\text{a}}$ ·°C^-1^); λ is the latent heat of vaporization (MJ·kg^-1^); *G_a_* and *G_s_* are air conductivity and canopy stomatal conductivity, respectively, where G_a_ (mol·m^-2^·s^-1^) can be quantified by the method proposed by Allen et al. (1998). The corresponding measurement and canopy heights for each site are shown in **Table 1**.

#### 2.3.2 TL B-B model

The B-B model (Ball et al., 1987), which originally estimated g_s_ based on leaf photosynthesis rates, was also used to estimate G_s_ in vegetated areas (Luo et al., 2018). Li et al. (2019) proposed a new G_s_ model for estimating g_s_ of sunlit and shaded leaves (Eq. (2)):


(2) $$G_{s}=g_{sh}\cdot \frac{GPP_{shaded}\cdot RH}{C_{a}}+g_{su}\cdot \frac{GPP_{sunlit}\cdot RH}{C_{a}}+G_{s,min}$$

where *g_sh_* and *g_su_* are empirical parameters of photosynthetic rate sensitivity for shaded and sunlit leaves, respectively, ranging from 0-60 (Li et al., 2019); *GPP_sunlit_* and *GPP_shaded_* (umol CO_2_·m^-2^·s^-1^) are photosynthetic rates of sunlit and shaded leaves, respectively; *RH* is the canopy surface relative humidity; *Ca* (umol CO_2_·mol^-1^) is the concentration of carbon dioxide in air; and *G_s,min_* (= 0.001mol·m^-2^·s^-1^) is the surface conductance of soil evaporative.

#### 2.3.3 TL-LUE model

Initially, He et al. (2013) developed a TL-LUE model based on the MOD17 algorithm, which divides the tree canopy into sunlit leaves and shaded leaves and calculates the respective GPP considering the differences in light absorption and LUE between the two groups of leaves. Similar to the MOD17 algorithm, the TL-LUE model only captures the constraint of low temperature and ignores the effect of high temperature on GPP. The modified TL-LUE model integrates the temperature scalar from the Terrestrial Ecosystem Model (TEM) to describe the effects of low and high temperatures on GPP (Raich et al., 1991; Li et al., 2019). The main algorithms is formulated as Eq. (3-5):


(3) $$GPP=GPP_{sunlit}+GPP_{shaded}$$

(4) $$GPP_{sunlit}=\epsilon_{msu}\cdot APAR_{sunlit}\cdot f\left(VPD\right)\cdot f\left(T\right)$$

(5) $$GPP_{shaded}=\epsilon_{msh}\cdot APAR_{shaded}\cdot f\left(VPD\right)\cdot f\left(T\right)$$

(6) $$APAR_{sunlit}=(1-\alpha )\times [PAR_{dir}\times \frac{\cos\beta }{\cos\theta }+\frac{PAR_{dir}-PAR_{dif,u}}{LAI}+C]\times LAI_{sunlit}$$

(7) $$APAR_{shaded}=(1-\alpha )\times [\frac{PAR_{dif}-PAR_{dif,u}}{LAI}+C]\times LAI_{shaded}$$

(8) $$LAI_{sunlit}=2\cos\left(\theta \right)\times \left[1-\exp\left(-\frac{0.5\Omega \times LAI}{\cos\left(\theta \right)}\right)\right]$$

(9) $$LAI_{shaded}=LAI_{tot}-LAI_{sunlit}$$

where *ϵ_msu_* and *ϵ_msh_* are the maximum LUE for sunlit and shaded leaves, ranging 0.34-1.50 and 2.71-4.79 gC/MJ, respectively (Bi et al., 2022); the subscripted “sunlit” and “shaded” denote the sunlit and shaded leaves, respectively; *f(VPD)* and *f(T)* are the limiting functions. *PAR_dir_ and PAR_dif_* denote the direct and diffuse photosynthetically active radiation (PAR), and *PAR_dif,u_ denotes PAR_dif_* under the canopy; *α*, *β*, *θ*, *C* and *Ω* denote the canopy albedo, leaf angle, solar zenith angle, contribution of multiple scattering of direct radiation per unit leaf area and the clumping index, respectively. The details of the equations and parameters are described in He et al. (2013) and Li et al. (2019).

### 2.4 Identification of the seasons

Three seasons were identified to present the temporal variability of the empirical parameters in the TL-LUE and TL B-B models. The seasonal thresholds of temperature were determined following the method of Park et al. (2018), which are the 25th and 75th percentiles of the average daytime temperature over the valid years of flux data for each of the study sites. Winter was defined as the days when the temperature was below the 25th percentile threshold, while summer was the period when the temperature was above the 75th percentile threshold. Spring and autumn were defined as the transitional periods between winter and summer, with increasing and decreasing temperatures, respectively.

### 2.5 Parameterization and evaluation

The key parameters in the TL-LUE model (ϵ_msu_ and ϵ_msh_) and TL B-B model (g_sh_ and g_su_) needed to be calibrated for each site under two parametrization schemes: (1) calibrated by all of the data during the entire growing season (from spring to autumn) of valid years (termed EGS), and (2) calibrated by the season-specific data of valid years (termed SEA). The shuffled complex evolution method of the University of Arizona (SCE-UA) was applied for the calibration, and the performance was evaluated using the agreement index (d, Eq. (10)) (Zhou et al., 2016b):


(10) $$d=1-\frac{\sum_{i=1}^{N}{\left(P_{i}-O_{i}\right)}^{2}}{\sum_{i=1}^{N}{\left(\left||P_{i}-\bar{O}\right|+\left||O_{i}-\bar{O}\right|\right)}^{2}}$$

where *N* is the total number of simulated experimental data points; *P_i_* and *O_i_* represent the predicted and observed values, respectively; $\bar{\text{P}}$ and $\bar{\text{O}}$ is the average of predicted and observed values for all experimental data points. Additionally, the estimated GPP, G_s_ and T_c_ using the two parametrized schemes were evaluated against the measured data using three indicators, i.e., the Akaike information criterion (AIC), root-mean square error (RMSE) and correlation coefficient (r).

## 3 Results

### 3.1 Calculations of APAR

**Figure 3:**
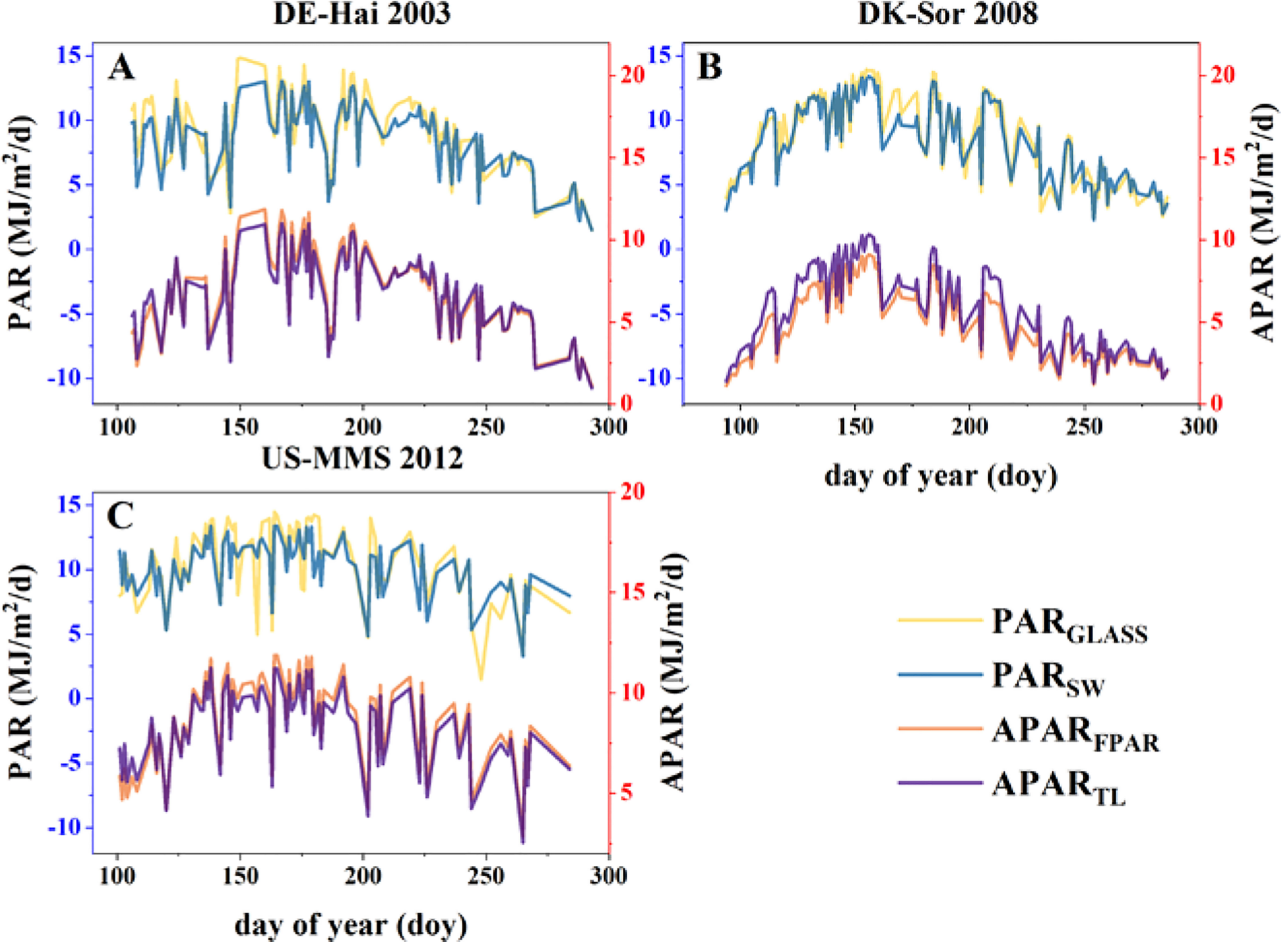
Intra-annual variability of daily photosynthetically active radiation (PAR) obtained from GLASS and flux data (SW), and absorbed PAR (APAR) calculated by the fraction of APAR (FPAR) and the two-leaf (TL) scheme at the study sites of **(A)** DE-Hai, **(B)** DK-Sor and **(C)** US-MMS.

The accuracy of calculated APAR is the key to estimate GPP and Tc, which depends on the precise of photosynthetically active radiation (PAR) data and FPAR. On the one hand, PAR_SW_, the shortwave incident radiation (“SW_IN_F”) multiplied by 0.45, is compared with the GLASS PAR product (PAR_GLASS_). The two PAR data sets showed a similar temporal variability (**Figure 3**) with a high r (0.91 ± 0.04) and a low RMSE (1.31 ± 0.22 MJ·m^-2^·d^-1^). On the other hand, APAR_FPAR_ was calculated by multiplying PAR_SW_ by GLASS FPAR. The APAR of sunlit and shaded leaves calculated under the TL scheme were summed to obtain the total APAR (APAR_TL_). Comparing the two APAR data, the results indicated a high agreement, with a strong r (> 0.96) and low RMSE (< 0.82 MJ·m^-2^·d^-1^). These findings indicate that both PAR calculated using the flux tower shortwave incident radiation and APAR calculated under the TL scheme showed a good accuracy for further analyses.

### 3.2 Estimations of GPP

**Figure 4:**
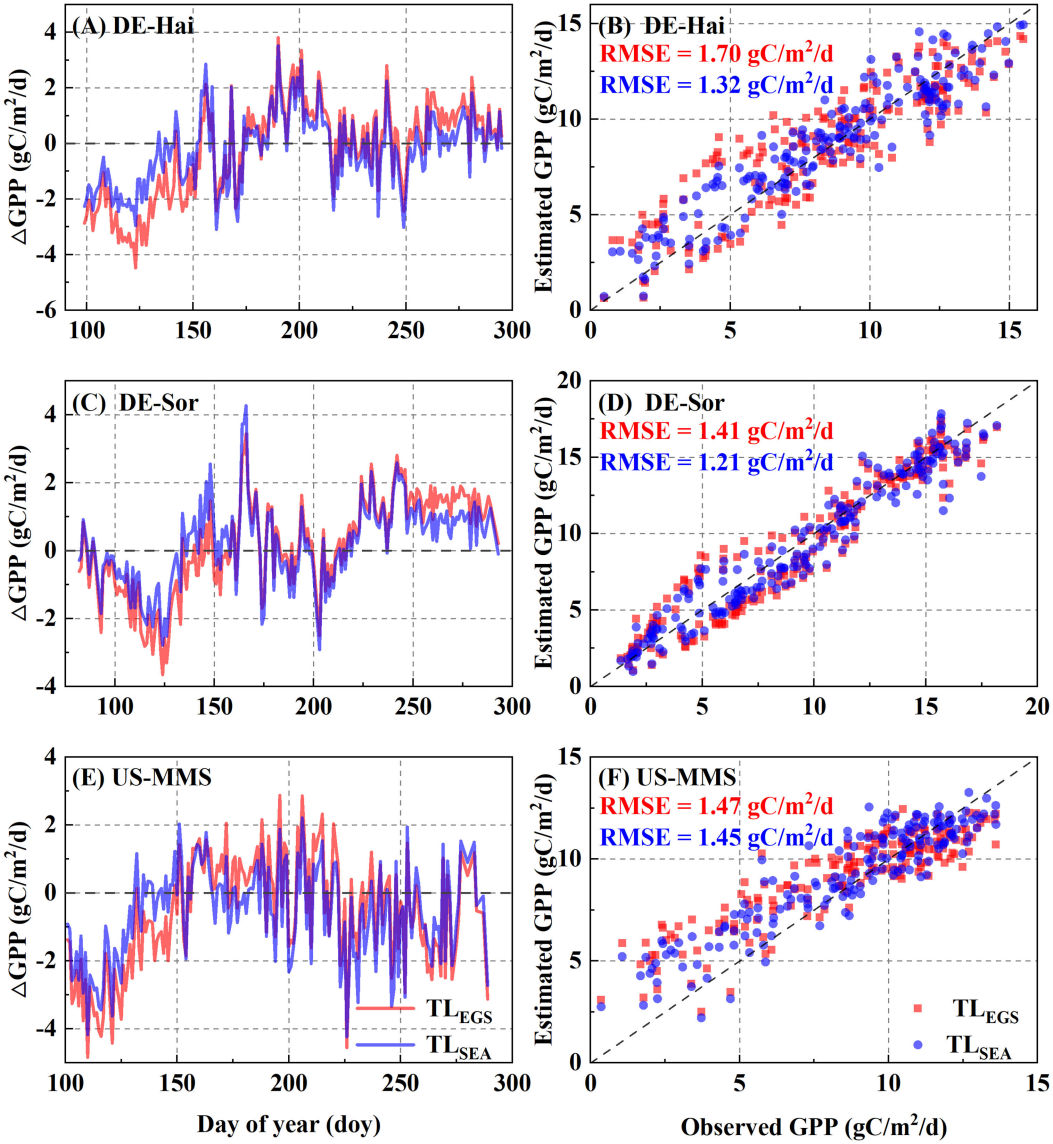
Comparisons between daily gross primary productivity (GPP) derived from the flux observations (FLUX) and the two-leaf-based (TL) simulations with the two parametrization schemes. EGS and SEA denote entire growth season-based fixed parametrization and season-specific dynamic parametrization, respectively. The left panels **(A, C, E)** show the inner-annual variabilities of the difference between observed and estimated GPP. The right panels **(B, D, F)** show the RMSE between estimated and observed GPP.

The maximum LUE (ϵ_msu_ and ϵ_msh_) of the representative sunlit and shaded leaves under the two parameterization schemes for each of the study sites are presented in **Table 2**. The results showed a significantly seasonal difference in ϵ_msu_ with a minimum in spring (0.50 ± 0.28 gC/MJ) and maximum in summer (0.78 ± 0.33 gC/MJ). The daily GPP time series estimated with the two parameterization scheme models are shown in **Figure 4**. Overall, the estimated GPP under the two schemes were generally consistent with the field observations and well captured the temporal variation of GPP at each study site. The statistical results are shown in **Table 3**. It shows the model with SEA better simulated GPP with a higher r (0.85 ± 0.08) than that with EGS (0.81 ± 0.09). Moreover, in the calibration groups, the average RMSE for the SEA model (2.10 ± 0.29 gC·m^-2^·d^-1^) was 8.2 ± 1.3% lower than that for the EGS model (2.28 ± 0.31 gC·m^-2^·d^-1^) across the sites. Meanwhile a lower AIC value of the SEA model (AIC_SEA_ = 731.37 ± 132.30) indicated that the seasonal dynamic parameters could improve the GPP estimation. The better performances of the SEA were also observed in the validation group. Across all sites, the average RMSE of the SEA model (2.11 ± 0.29 gC·m^-2^·d^-1^) was 8.0 ± 1.1% lower than that of the EGS model (2.29 ± 0.30 gC·m^-2^·d^-1^), and the average value of r of the SEA model (0.84 ± 0.08) was 3.7 ± 1.5% higher than that of the EGS model (0.81 ± 0.09). Furthermore, the model with SEA was able to better capture the seasonal variation of GPP and effectively reduce the overestimation of GPP in spring and autumn. The greatest decrease in RMSE was observed in spring at the DE-Hai site (21.4%) and in autumn at the DK-Sor site (27.7%).

**Table 2:** Average (standard deviation) of the calibrated maximum light use efficiency (ϵ_msu_ and ϵ_msh_ (gC·MJ^-1^) for sunlit and shaded leaves, respectively) under the two parameterized schemes.

Site ID	ϵ_msu_		ϵ_msh_		Seasons
	EGS	SEA	EGS	SEA	
DE-Hai	0.34 (0.000065)	0.34 (0.000022)	4.16 (0.028)	3.34 (0.067)	Spring
		0.46 (0.064)		4.12 (0.11)	Summer
		0.36 (0.039)		4.74 (0.061)	Autumn
DK-Sor	1.05 (0.012)	0.83 (0.027)	4.79 (0.00028)	4.79 (0.00032)	Spring
		1.12 (0.013)		4.79 (0.00026)	Summer
		1.50 (0.000085)		4.79 (0.00017)	Autumn
US-MMS	0.41 (0.051)	0.34 (0.0027)	3.47 (0.10)	3.10 (0.046)	Spring
		0.75 (0.094)		3.05 (0.18)	Summer
		0.49 (0.11)		2.99 (0.24)	Autumn

EGS and SEA denote the entire growth season-based fixed parametrization and season-specific dynamic parametrization, respectively.

**Table 3:** Calibration and validation performances of the two-leaf light use efficiency model with the two parameterized schemes in estimating gross primary productivity (GPP).

Site ID		EGS				SEA			
		AIC	RMSE	r	d	AIC	RMSE	r	d
DE-Hai	Calibration	851.81 (15.46)	2.44 (0.04)	0.79 (0.01)	0.88 (0.01)	763.95 (17.41)	2.21 (0.04)	0.83 (0.01)	0.90 (0.005)
DK-Sor		660.76 (15.99)	1.93 (0.03)	0.91 (0.004)	0.96 (0.002)	581.40 (18.19)	1.77 (0.03)	0.93 (0.003)	0.96 (0.002)
US-MMS		914.82 (19.67)	2.48 (0.05)	0.74 (0.01)	0.81 (0.01)	848.76 (21.50)	2.31 (0.05)	0.78 (0.01)	0.85 (0.01)
DE-Hai	Validation	369.36 (15.39)	2.44 (0.09)	0.79 (0.02)	0.88 (0.01)	339.15 (16.72)	2.22 (0.09)	0.83 (0.02)	0.90 (0.01)
DK-Sor		290.20 (16.43)	1.94 (0.07)	0.91 (0.01)	0.95 (0.005)	261.88 (17.61)	1.78 (0.07)	0.93 (0.01)	0.96 (0.004)
US-MMS		399.14 (17.96)	2.49 (0.10)	0.74 (0.03)	0.81 (0.02)	377.71 (18.62)	2.32 (0.10)	0.77 (0.02)	0.85 (0.01)

EGS and SEA denote the entire growth season-based fixed parametrization and season-specific dynamic parametrization, respectively. AIC, RMSE (gC·m^-2^·d^-1^), r and d indicate the Akaike information criterion, root-mean square error, correlation coefficient, and agreement index, respectively. The average (standard deviation) values of the evaluation indicators from the randomized experiments are shown in the table.

### 3.3 Estimations of G_s_

The parameters g_sh_ and g_su_ calibrated using the two parameterization schemes are displayed in **Table 4**. Obviously seasonal variations in g_sh_ and g_su_ were observed at each study site, with the lower values in summer and slightly higher values in spring and autumn. Overall, the seasonal dynamic parameters g_sh_ and g_su_ of the SEA scheme improved the estimations of G_s_ relative to the fixed parameters of the EGS scheme (**Table 5**). In the calibration group, the average RMSE of the G_s_ estimated with the SEA scheme (0.083 ± 0.067 mol·m^-2^·s^-1^) was 6.5 ± 4.6% lower than that estimated with the EGS scheme (0.093 ± 0.076 mol·m^-2^·s^-1^), as well as the AIC values (AIC_EGS_ = -2517.62 ± 690.81 vs. AIC_SEA_ = -2568.11 ± 740.32). In the validation group, the SEA model also performed better than the EGS model, with an average RMSE of 0.086 ± 0.066 mol·m^-2^·s^-1^ and 0.094 ± 0.075 mol·m^-2^·s^-1^, respectively, across the study site. However, when considering the AIC, the SEA model (-1162.79) did not perform as well as the EGS model (-1172.67) at DE-Hai. Although the r of the SEA model was lower than that of the EGS model at the DK-Sor site, the TL B-B model with the dynamic parameterization effectively reduced the errors of estimated G_s_ compared to that with fixed parameterization.

**Table 4:** Average (standard deviation) of the calibrated slope parameter of the Ball-Berry model (g_su_ and g_sh_ for sunlit and shaded leaves, respectively) under the two parameterized schemes.

Site ID	g_sh_		g_su_		Seasons
	EGA	SEA	EGA	SEA	
DE-Hai	7.15 (0.066)	8.30 (0.15)	0.0023 (0.0025)	0.0015 (0.0017)	Spring
		6.98 (0.19)		0.0022 (0.0026)	Summer
		7.95 (0.25)		0.33 (0.95)	Autumn
DK-Sor	18.51 (0.24)	15.70 (0.51)	0.0012 (0.0014)	0.63 (0.98)	Spring
		15.60 (1.40)		0.00074 (0.0010)	Summer
		39.68 (1.70)		0.00051 (0.00065)	Autumn
US-MMS	6.08 (0.18)	7.32 (0.20)	0.094 (0.23)	0.28 (0.61)	Spring
		6.59 (0.39)		0.16 (0.24)	Summer
		7.50 (0.59)		0.043 (0.22)	Autumn

EGS and SEA denote the entire growth season-based fixed parametrization and season-specific dynamic parametrization, respectively.

**Table 5:** Calibration and validation performances of the two-leaf Ball-Berry model with the two parameterized schemes in estimating canopy conductance (G_s_).

Site ID		EGA				SEA			
		AIC	RMSE	r	d	AIC	RMSE	r	d
DE-Hai	Calibration	-2690.09 (20.70)	0.06 (0.001)	0.65 (0.01)	0.80 (0.01)	-2735.78 (21.93)	0.05 (0.001)	0.69 (0.01)	0.82 (0.01)
DK-Sor		-1706.28 (26.59)	0.18 (0.005)	0.40 (0.02)	0.64 (0.01)	-1808.90 (59.38)	0.16 (0.01)	0.51 (0.04)	0.71 (0.03)
US-MMS		-3156.48 (20.77)	0.04 (0.0009)	0.68 (0.01)	0.82 (0.01)	-3159.65 (20.81)	0.04 (0.009)	0.68 (0.01)	0.82 (0.01)
DE-Hai	Validation	-1172.67 (22.03)	0.06 (0.003)	0.69 (0.03)	0.82 (0.02)	-1162.79 (20.53)	0.06 (0.003)	0.69 (0.03)	0.82 (0.02)
DK-Sor		-731.41 (25.37)	0.18 (0.01)	0.39 (0.05)	0.64 (0.03)	-762.90 (25.48)	0.16 (0.01)	0.51 (0.04)	0.71 (0.03)
US-MMS		-1339.47 (23.41)	0.04 (0.002)	0.68 (0.03)	0.79 (0.02)	-1349.22 (19.54)	0.04 (0.002)	0.68 (0.03)	0.82 (0.02)

EGS and SEA denote the entire growth season-based fixed parametrization and season-specific dynamic parametrization, respectively. AIC, RMSE (mol·m^-2^·s^-1^), r and d indicate the Akaike information criterion, root-mean square error, correlation coefficient, and agreement index respectively. The average (standard deviation) values of the evaluation indicators from the randomized experiments are shown in the table.

### 3.4 Estimations of T_c_

**Figure 5:**
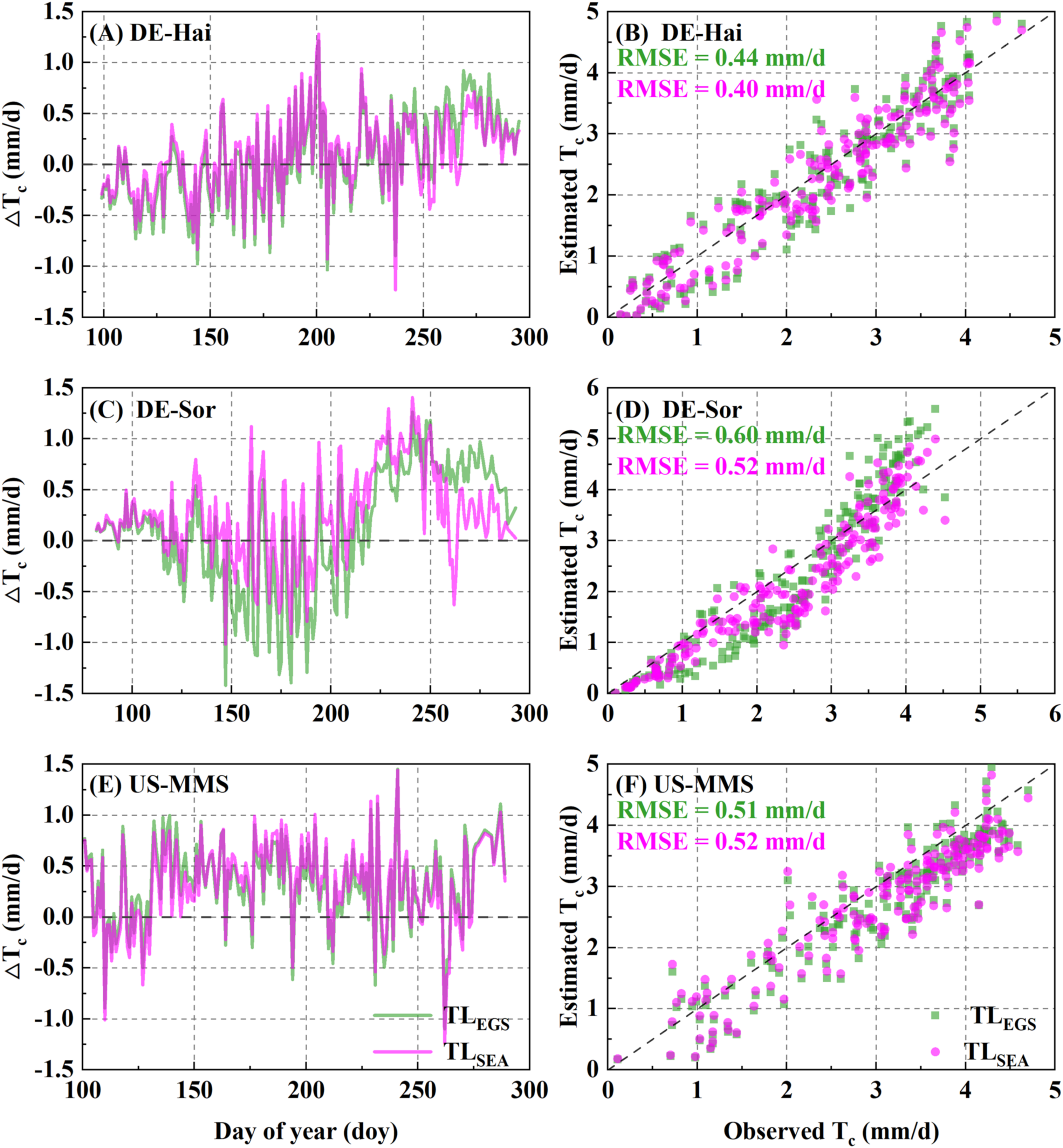
Comparisons between daily transpiration (T_c_) derived from the flux observations (FLUX) and the two-leaf-based simulations with the two parametrization schemes. EGS and SEA denote entire growth season-based fixed parametrization and season-specific dynamic parametrization, respectively. The left panels **(A, C, E)** show the inner-annual variabilities of the difference between observed and estimated T_c_. The right panels **(B, D, F)** show the RMSE between estimated and observed T_c_.

T_c_ was estimated using the P-M model combined with the two parameterization schemes. **Figure 5** shows the daily T_c_ estimations with the corresponding measured values. The results of the two schemes were generally consistent with the observed values of T_c_ and well captured the temporal variation of that. The statistical results (**Table 6**) showed improvements of the SEA-based T_c_ estimation relative to the EGS-based one. In the calibration group, the average RMSE of the SEA model (0.75 ± 0.06 mm·d^-1^) was 3.7 ± 4.4% lower than that of the EGS model (0.78 ± 0.07 mm·d^-1^) across the study sites. While for the US-MMS site, the EGS model outperformed. Similarly, in the validation group, the SEA model (with an average RMSE of 0.76 ± 0.07 mm·d^-1^) performed better than the EGS model (with an average RMSE of 0.82 ± 0.12 mm·d^-1^) at all of the study sites except DE-Hai. However, the correlations between the observed and estimated T_c_ of the SEA model were slightly lower than that of the EGS model. Moreover, the improvement of the SEA model was mainly found in spring and autumn across the sites, with a lower RMSE of 7.9 ± 2.8% and 17.6 ± 16.8% relative to the EGS model, respectively. However, the improvement in summer was not significant, with only a 0.7% and 0.6% reduction at DE-Hai and DK-Sor, respectively, while even a 4.4% increase at the US-MMS site.

**Table 6:** Calibration and validation performances of the two-leaf Penman-Monteith model with the two parameterized schemes in estimating transpiration (T_c_).

Site ID		EGA				SEA			
		AIC	RMSE	r	d	AIC	RMSE	r	d
DE-Hai	Calibration	-313.39 (19.13)	0.71 (0.01)	0.86 (0.01)	0.92 (0.004)	-338.11 (19.17)	0.68 (0.01)	0.87 (0.01)	0.93 (0.003)
DK-Sor		-155.04 (17.56)	0.85 (0.01)	0.90 (0.0040)	0.91 (0.0029)	-220.64 (47.96)	0.78 (0.04)	0.88 (0.01)	0.92 (0.01)
US-MMS		-234.90 (15.32)	0.78 (0.01)	0.82 (0.01)	0.89 (0.005)	-208.36 (15.38)	0.79 (0.01)	0.81 (0.01)	0.88 (0.01)
DE-Hai	Validation	-148.06 (18.78)	0.68 (0.03)	0.88 (0.01)	0.93 (0.01)	-130.17 (18.92)	0.68 (0.03)	0.87 (0.01)	0.93 (0.01)
DK-Sor		-61.91 (18.94)	0.85 (0.04)	0.90 (0.01)	0.91 (0.01)	-76.83 (24.72)	0.79 (0.05)	0.88 (0.02)	0.92 (0.02)
US-MMS		-27.72 (26.79)	0.92 (0.06)	0.82 (0.02)	0.84 (0.02)	-73.42 (16.92)	0.80 (0.03)	0.81 (0.02)	0.88 (0.01)

EGS and SEA denote entire growth season-based fixed parametrization and season-specific dynamic parametrization, respectively. AIC, RMSE (mm·d^-1^), r and d indicate the Akaike information criterion, root-mean square error, correlation coefficient, and agreement index respectively. The average (standard deviation) values of the evaluation indicators from the randomized experiments are shown in the table.

## 4 Discussion

Improving the accuracy of T_c_ estimates requires refinement of the key parameters in the photosynthesis and g_s_-A models. Previous studies have demonstrated that photosynthetic and hydraulic indicators exhibit significant seasonal variability. However, the empirical parameters (e.g., ϵ_msu_ and ϵ_msh_ of the TL-LUE model, and g_su_ and g_sh_ in the TL B-B model) are commonly set to biome-specific constants in current GPP and G_s_ modelling (Miner et al., 2017; Pei et al., 2022). In this study, we hypothesize that considering the seasonal dynamics of the physiological parameters will improve T_c_ estimations. To test this hypothesis, we assume that these parameters are subject to seasonal variability in both the TL-LUE and B-B models.

Our results showed that sunlit leaves exhibited a lower maximum LUE (ϵ_msu_) relative to shaded leaves (ϵ_msh_) at both the entire growing season and each season. This is because sunlit leaves are able to absorb both direct and scattered radiation, but are susceptible to light saturation in instances of intense radiation. In contrast, shaded leaves only receive scattered radiation (including multiple scattering of direct solar radiation within the canopy), and their absorbed radiation intensity is typically between the light compensation and saturation points, resulting in a higher LUE (Mercado et al., 2009; Oliphant et al., 2011). Similarly, an obviously higher value of g_sh_ relative to g_sh_ was also observed in each season, which is in line with Li et al. (2019). Additionally, previous studies reported that the BL scheme may induce negative biases in estimated T_c_ compared to the TL scheme when the LAI was large (Chen and Liu, 2020), which also supported by our results. This is because TL scheme performs better in the calculation of the portions of light-saturated and light-unsaturated leaves in the canopy (Luo et al., 2018).

Moreover, a seasonality of ϵ_msu_ was found in this study, with the lowest value in spring and the highest in summer. Similarly, the results showed an explicitly seasonal variation in g_sh_ and g_su_, of which the average was the lowest in summer and slightly higher in spring and autumn. These are consistent with previous studies that shown temporal variation in optimal LUE and the slope parameter of g_s_-A models (Wolf et al., 2006; Chen et al., 2011; Ryu et al., 2011; Franks et al., 2017; Jin et al., 2022). We infer that leaf phenology and environmental variables mutually determine the seasonal variability of the physiological parameters. Previous studies have established that leaf age plays a significant role in photosynthesis in temperate zones, showing a highest photosynthetic capacity and intrinsic water use efficiency of mature leaves (Wilson et al., 2001; Pantin et al., 2012; Albert et al., 2018). Additionally, plant photosynthetic and hydraulic traits can acclimate the environment in coordination over weeks to months according to the evolutionary optimality hypothesis (Wang et al., 2017).

Furthermore, the dynamic parameterization scheme (i.e., SEA) showed have a significant improvement in the GPP and G_s_ estimation compared to the fixed parameterization scheme (i.e., EGS) in each site. Although a better performance of SEA in both the calibration and validation of T_c_ was seen only in DK-Sor, the average error of estimated T_c_ using SEA was lower than that using EGS across the study sites. Previous studies on improvements of empirical or process models with dynamic parametrizations also supported our results. For example, Zhang et al. (2010) established the relationship between the optimal G_s_ and NDVI using a sigmoid response function by which the estimation of canopy conductance was remarkably improved. In addition, a recent study of Jin et al. (2022) estimated G_s_ by incorporating a LAI-based dynamic parametrization of G_1_ (the slope parameter of the optimality-based unified stomatal optimization model), showing a significantly increasing accuracy of modelling T_c_ at both daily and seasonal scales. Some limitations should be noted in our present work. The growing season and sub-periods were identified by multi-year average temperatures (Park et al., 2018) rather than plant phenology. Hence, this study ignored variabilities of phenology with climatic variables (Wang et al., 2020; Lin et al., 2022), which may directly and/or indirectly impact the physiological parameters in the seasons (Richardson et al., 2013). Besides, the T_c_ data were derived from the observed latent heat fluxes by an uWUE-based method (Zhou et al., 2015, 2016a) and ignored the effects of water fluxes from understory plants, which may lead to errors in the model calibration and validation. Additional direct measurements of T_c_, e.g. the lysimeter and isotope measurements, should be take into account to reduce the uncertainties in further studies. Nevertheless, the achievements of this study highlight the importance of considering seasonal variation in key parameters in T_c_ modelling, and the methods proposed here are applicable to species of which leaf development or canopy activity shows an explicit seasonal variability in a year.

## 5 Conclusion

This study highlighted the importance of incorporating seasonal variations in the key parameters of two-leaf conductance-photosynthesis models to improve estimations of T_c_. The results indicate that ϵ_msu_ showed a clear seasonal pattern, with the highest value in summer. Meanwhile, both g_su_ and g_sh_ appeared slightly higher values in spring and autumn than that in summer. Relative to the fixed parameterized scheme, our findings suggested that applying the seasonal dynamic parameterization can effectively reduce errors in the simulation of GPP and T_c_ at a daily scale. We therefore recommend the consideration of seasonal dynamic parameterizations in ecosystem models to more accurately simulate carbon and water fluxes under a changing climate.

## Data availability statement

The original contributions presented in the study are included in the article/supplementary material. Further inquiries can be directed to the corresponding authors.

## Author contributions

Conceptualization, JJ and YW. Data curation and validation, YL. Methodology, software and formal analysis, YL and JJ. Project administration, JJ and YW. Writing—original draft, JJ and YL. Writing—review and editing, WH, YC, FZ, YW, XF, LH, BY and LR. All authors have read and agreed to the published version of the manuscript.

## References

[B1] Albert L. P. Wu J. Prohaska N. Camargo P. D. Huxman T. E. Tribuzy E. S.. (2018). Age-dependent leaf physiology and consequences for crown-scale carbon uptake during the dry season in an Amazon evergreen forest. New Phytol. 219 (3), 870. doi: 10.1111/nph.15340 29502356

[B2] Allen R. G. Pereira L. S. Raes D. Smith M. (1998). “Crop evapotranspiration: guidelines for computing crop requirements,” in FAO irrigation and drainage paper, vol. 56. (Rome: Food and Agricultural Organization of the U.N).

[B3] Ball J. T. Woodrow I. E. Berry J. A. (1987). “A model predicting stomatal conductance and its contribution to the control of photosynthesis under different environmental conditions,” in Progress in photosynthesis research (Dordrecht: Springer Science+Business Media, London). New York. doi: 10.1007/978-94-017-0519-6_48

[B4] Bi W. J. He W. Zhou Y. L. Ju W. M. Liu Y. B. Liu Y. (2022). A global 0.05° dataset for gross primary production of sunlit and shaded vegetation canopies from 1992 to 2020. Sci. Data 9 (1), 393. doi: 10.1038/s41597-022-01309-2 35577806 PMC9110750

[B5] Chavana-Bryant C. Malhi Y. Anastasiou A. Enquist B. J. Cosio E. G. Keenan T. F. (2019). Leaf age effects on the spectral predictability of leaf traits in Amazonian canopy trees. Sci. Total Environ. 666, 1301–1315. doi: 10.1016/J.SCITOTENV.2019.01.379 30970495

[B6] Chen J. Jönsson P. Tamura M. Gu Z. H. Matsushita B. Eklundh L. (2004). A simple method for reconstructing a high-quality NDVI time-series dataset based on the savitzky-golay filter. Remote Sens. Environ. 91 (3-4), 332–344. doi: 10.1016/j.rse.2004.01.009

[B7] Chen J. M. Liu J. (2020). Evolution of evapotranspiration models using thermal and shortwave remote sensing data. Remote Sens. Environ. 237, 111594. doi: 10.1016/j.rse.2019.111594

[B8] Chen J. M. Liu J. Cihlar J. Goulden M. L. (1999). Daily canopy photosynthesis model through temporal and spatial sealing for remote sensing applications. Ecol. Model. 124 (2/3), 99–119. doi: 10.1016/S0304-3800(99)00138-6

[B9] Chen T. van der Werf G. R. Dolman A. J. Groenendijk M. (2011). Evaluation of cropland maximum light use efficiency using eddy flux measurements in north America and Europe. Geophys. Res. Lett. 38, L14707. doi: 10.1029/2011GL047533

[B10] Dai Y. Dickinson R. E. Wang Y. P. (2004). A two-big-leaf model for canopy temperature, photosynthesis, and stomatal conductance. J. Climate 17 (12), 2281–2299. doi: 10.1175/1520-0442(2004)017<2281:ATBMFC>2.0.CO;2

[B11] Ding Z. Zheng H. Wang J. O’Connor P. Li C. Chen X.. (2022). Integrating top-down and bottom-up approaches improves practicality and efficiency of large-scale ecological restoration planning: insights from a social-ecological system. Engineering doi: 10.1016/j.eng.2022.08.008

[B12] Franks P. J. Berry J. A. Lombardozzi D. L. Bonan G. B. (2017). Stomatal function across temporal and spatial scales: deep-time trends, land-atmosphere coupling and global models. Plant Physiol. 174 (2), 583–602. doi: 10.1104/pp.16.01293 28446638 PMC5462067

[B13] He M. Z. Ju W. M. Zhou Y. L. Chen J. M. He H. L. Wang S. Q.. (2013). Development of a two-leaf light use efficiency model for improving the calculation of terrestrial gross primary productivity. Agric. For. Meteorol. 173, 28–39. doi: 10.1016/j.agrformet.2012.11.005

[B14] Hetherington A. M. Woodward F. I. (2003). The role of stomata in sensing and driving environmental change. Nature 424 (6951), 901–908. doi: 10.1038/nature01843 12931178

[B15] Jasechko S. Sharp Z. D. Gibson J. J. Birks S. J. Yi Y. Fawcett P. J. (2013). Terrestrial water fluxes dominated by transpiration. Nature 496 (7445), 347–350. doi: 10.1038/nature12045 23552893

[B16] Jin J. Yan T. Wang H. Ma X. L. He M. Z. Wang Y.. (2022). Improved modeling of canopy transpiration for temperate forests by incorporating a LAI-based dynamic parametrization scheme of stomatal slope. Agric. For. Meteorol. 326, 109157. doi: 10.1016/j.agrformet.2022.02.039

[B17] Katul G. Manzoni S. Palmroth S. Oren R. (2010). A stomatal optimization theory to describe the effects of atmospheric CO2 on leaf photosynthesis and transpiration. Ann. Bot. 105 (3), 431–442. doi: 10.1093/aob/mcp292 19995810 PMC2826246

[B18] Kimball J. S. Thornton P. E. White M. A. Running S. W. (1997). Simulating forest productivity and surface-atmosphere carbon exchange in the BOREAS study region. Tree Physiol. 17 (8/9), 589–599. doi: 10.1093/treephys/17.8-9.589 14759832

[B19] Knohl A. Schulze E.D. Kolle O. Buchmann N. (2003). Large carbon uptake by an unmanaged 250-year-old deciduous forest in Central Germany. Agric. For. Meteorol. 118 (3-4), 151–167. doi: 10.1016/S0168-1923(03)00115-1

[B20] Lawrence D. M. Oleson K. W. Flanner M. G. Thornton P. E. Swenson S. C. Lawrence P. J.. (2011). Parameterization improvements and functional and structural advances in version 4 of the community land model. J. Adv. Model. Earth Syst. 3 (1), M03001. doi: 10.1029/2011MS00045

[B21] Leuning R. Kelliher F. M. Pury D. G. G. D. Schulze E. D. (1995). Leaf nitrogen, photosynthesis, conductance and transpiration: scaling from leaves to canopies. Plant Cell Environ. 18 (10), 1183–1200. doi: 10.1111/j.1365-3040.1995.tb00332.x

[B22] Li J. Ju W. He W. Wang H. Zhou Y. Xu M. (2019). An algorithm differentiating sunlit and shaded leaves for improving canopy conductance and evapotranspiration estimates. J. Geophys. Res. Biogeosci. 124 (4), 807–824. doi: 10.1029/2018JG005038

[B23] Li Y. Piao S. L. Li L. Z. X. Chen A. P. Wang. X. H. Ciais P.. (2018). Divergent hydrological response to large-scale afforestation and vegetation greening in China. Sci. Adv. 4, eaar4182. doi: 10.1126/sciadv.aar4182 29750196 PMC5942916

[B24] Liang S. Cheng J. Jia K. Jiang B. Zhou J. (2021). The global land surface satellite (GLASS) product suite. B. A. Meteorol. Soc. 102 (2), E323–E337. doi: 10.1175/BAMS-D-20-0151.1

[B25] Lin S. Wang H. Ge Q. Hu Z. (2022). Effects of chilling on heat requirement of spring phenology vary between years. Agric. For. Meteorol. 312, 108718. doi: 10.1016/j.agrformet.2021.108718

[B26] Liu Y. B. Qiu G. Y. Zhang H. S. Yang Y. H. Zhang Y. S. Wang Q.. (2022). Shifting from homogeneous to heterogeneous surfaces in estimating terrestrial evapotranspiration: review and perspectives. Sci. China Earth Sci. 65 (2), 197–214. doi: 10.1007/s11430-020-9834-y

[B27] Luo X. Chen J. M. Liu J. Black T. A. Croft H. Staebler R.. (2018). Comparison of big-leaf, two-big-leaf, and two-leaf upscaling schemes for evapotranspiration estimation using coupled carbon-water modeling. J. Geophys. Res. Biogeosci. 123 (1), 207–225. doi: 10.1002/2017JG003831

[B28] Medlyn B. E. Duursma R. A. Eamus D. Ellsworth D. S. Prentice I. C. Barton C. V. (2011). Reconciling the optimal and empirical approaches to modelling stomatal conductance. Glob. Chang. Biol. 17 (6), 2134–2144. doi: 10.1111/j.1365-2486.2010.02375.x

[B29] Mercado L. Bellouin N. Sitch S. Boucher O. Cox P. M. (2009). Impact of changes in diffuse radiation on the global land carbon sink. Nature 458 (7241), 1014–1U87. doi: 10.1038/nature07944 19396143

[B30] Miner G. L. Bauerle W. L. Baldocchi D. D. (2017). Estimating the sensitivity of stomatal conductance to photosynthesis: a review. Plant Cell Environ. 40 (7), 1214–1238. doi: 10.1111/pce.13023 27925232

[B31] Monteith J. L. Unsworth M. H. (2013). Principles of environmental physics (Amsterdam, Netherlands: Elsevier).

[B32] Mu Q. Z. Zhao M. S. Running S. W. (2011). Improvements to a MODIS global terrestrial evapotranspiration algorithm. Remote Sens. Environ. 115 (8), 1781–1800. doi: 10.1016/j.rse.2011.05.005

[B33] Oliphant A. J. Dragoni D. Deng B. Grimmond C. S. B. Schmid H. P. Scott S. L. (2011). The role of sky conditions on gross primary production in a mixed deciduous forest. Agric. For. Meteorol. 151 (7), 781–791. doi: 10.1016/j.agrformet.2011.01.013

[B34] Pantin F. Simonneau T. Muller B. (2012). Coming of leaf age: control of growth by hydraulics and metabolics during leaf ontogeny. New Phytol. 196 (2), 349–366. doi: 10.1111/j.1469-8137.2012.04273.x 22924516

[B35] Park B. J. Kim Y. H. Min S. K. Lim E. P. (2018). Anthropogenic and natural contributions to the lengthening of the summer season in the northern hemisphere. J. Climate 31 (17), 6803–6819. doi: 10.1175/JCLI-D-17-0388.1

[B36] Pei Y. Dong J. Zhang Y. Yuan W. Doughty R. Yang J.. (2022). Evolution of light use efficiency models: improvement, uncertainties, and implications. Agric. For. Meteorol. 317,108905. doi: 10.1016/j.agrformet.2022.108905

[B37] Pilegaard K. Ibrom A. Courtney M. S. Hummelshøj P. Jensen N. O. (2011). Increasing net CO2 uptake by a Danish beech forest during the period from 1996 to 2009. Agric. For. Meteorol. 151 (7), 934–946. doi: 10.1016/j.agrformet.2011.02.013

[B38] Raich J. W. Rastetter E. B. Melillo J. M. Kicklighter D. W. Steudler P. A. Peterson B. J. (1991). Potential net primary productivity in south America: application of a global model. Ecol. Appl. 1 (4), 399–429. doi: 10.2307/1940456 27755669

[B39] Reichstein M. Bahn M. Ciais P. Frank D. Mahecha M. D. Seneviratne S. I.. (2005). On the separation of net ecosystem exchange into assimilation and ecosystem respiration: review and improved algorithm. Global Change Biol. 11 (9), 1424–1439. doi: 10.1111/j.1365-2486.2005.001002.x

[B40] Richardson A. D. Keenan T. F. Migliavacca M. Ryu Y. Sonnentag O. Toomey M. (2013). Climate change, phenology, and phenological control of vegetation feedbacks to the climate system. Agric. For. Meteorol. 169, 156–173. doi: 10.1016/j.agrformet.2012.09.012

[B41] Ryu Y. Baldocchi D. D. Kobayashi H. Ingen C. V. Roupsard O. (2011). Integration of MODIS land and atmosphere products with a coupled-process model to estimate gross primary productivity and evapotranspiration from 1 km to global scales. Global Biogeochem. Cy. 25 (4), GB4017. doi: 10.1029/2010GB003903

[B42] Schmid H. P. Grimmond C.S. B. Cropley F. Offerle B. Su H. B. (2006). Measurements of CO2 and energy fluxes over a mixed hardwood forest in the mid-western United States. Agric. For. Meteorol. 103 (4), 357–374. doi: 10.1016/S0168-1923(00)00140-4

[B43] Sellers P. J. Berry J. A. Collatz G. J. Field C. B. Hall F. G. (1992). Canopy reflectance, photosynthesis, and transpiration. III. a reanalysis using improved leaf models and a new canopy integration scheme. Remote Sens. Environ. 42, 1878–2216. doi: 10.1016/0034-4257(92)90140-E

[B44] Shen M. G. Piao S. L. Jeong S. J. Zhou L. M. Zeng Z. Z. Ciais P.. (2015). Evaporative cooling over the Tibetan plateau induced by vegetation growth. P. Natl. Acad. Sci. U.S.A. 112 (30), 9299–9304. doi: 10.1073/pnas.1504418112 PMC4522821 26170316

[B45] Wang K. Dickinson R. E. (2012). A review of global terrestrial evapotranspiratio-n: observation, modelling, climatology, and climatic variability. Rev. Geophy. 50, RG2005. doi: 10.1029/2011RG000373

[B46] Wang Y. P. Leuning R. (1998). A two-leaf model for canopy conductance, photosynthesis and partitioning of available energy. I. Agric. For. Meteorol. 91 (1-2), 89–111. doi: 10.1016/S0168-1923(98)00010-4

[B47] Wang H. Prentice I. C. Keenan T. F. Davis T. W. Wright I. J. Cornwell W. K.. (2017). Towards a universal model for carbon dioxide uptake by plants. Nat. Plants 3, 734–741. doi: 10.1038/s41477-017-0006-8 29150690

[B48] Wang H. Wu C. Ciais P. Peñuelas J. Dai J. H. Fu Y.. (2020). Overestimation of the effect of climatic warming on spring phenology due to misrepresentation of chilling. Nat. Commun. 11, 4945. doi: 10.1038/s41467-020-18743-8 33009378 PMC7532433

[B49] Wilson K. B. Baldocchi D. D. Hanson P. J. (2001). Leaf age affects the seasonal pattern of photosynthetic capacity and net ecosystem exchange of carbon in a deciduous forest. Plant Cell Environ. 24, 571–583. doi: 10.1046/j.1365-3040.2001.00715.x

[B50] Wolf A. Akshalov K. Saliendra N. Johnson D. A. Laca E. A. (2006). Inverse estimation of vcmax, leaf area index, and the ball-berry parameter from carbon and energy fluxes. Geophys. Res. 111 (D8), D08S08. doi: 10.1029/2005JD006662

[B51] Xiao Z. Liang S. Sun R. Wang J. Jiang B. (2015). Estimating the fraction of absorbed photosynthetically active radiation from the MODIS data based GLASS leaf area index product. Remote Sens. Environ. 171, 105–117. doi: 10.1016/j.rse.2015.10.016

[B52] Xiao Z. Liang S. Wang J. Chen P. Song J. (2014). Use of general regression neural networks for generating the GLASS leaf area index product from time-series MODIS surface reflectance. IEEE Trans. Geosci. Remote Sens. 52 (1), 209–223. doi: 10.1109/TGRS.2013.2250861

[B53] Yebra M. Van Dijk A. Leuning R. Huete A. Guerschman J. P. (2013). Evaluation of optical remote sensing to estimate actual evapotranspiration and canopy conductance. Remote Sens. Environ. 129, 250–261. doi: 10.1016/j.rse.2012.09.037

[B54] Zhang Y. Joiner J. Alemohammad S. H. Zhou S. Gentine P. (2018). A global spatially contiguous solar-induced fluorescence (CSIF) dataset using neural networks. Biogeosciences 15 (19), 5779–5800. doi: 10.5194/bg-15-5779-2018

[B55] Zhang K. Kimball J. S. Nemani R. R. Running S. W. (2010). A continuous satellite-derived global record of land surface evapotranspiration from 1983 to 2006. Water Resour. Res. 46 (9), W09522. doi: 10.1029/2009WR008800

[B56] Zhang Y. Kong D. Gan R. Chiew F. H. S. Mcvicar T. R. Zhang Q.. (2019). Coupled estimation of 500 m and 8-day resolution global evapotranspiration and gross primary production in 2002-2017. Remote Sens. Environ. 222, 165–182. doi: 10.1016/j.rse.2018.12.031

[B57] Zhang K. Zhu G. Ma J. Yang Y. Shang S. Gu C. (2019). Parameter analysis and estimates for the MODIS evapotranspiration algorithm and multiscale verification. Water Resour. Res. 55 (3), 2211–2231. doi: 10.1029/2018WR023485

[B58] Zhou Y. L. Wu X. C. Ju W. M. Chen J. M. Wang S. Q. Wang H. M.. (2016b). Global parameterization and validation of a two-leaf light use efficiency model for predicting gross primary production across FLUXNET sites. J. Geophys. Res. Biogeosci. 121 (4), 1045–1072. doi: 10.1002/2015JG002964

[B59] Zhou S. Yu B. Huang Y. Wang G. (2015). Daily underlying water use efficiency for AmeriFlux sites. J. Geophys. Res.: Biogeosci. 120 (5), 887–902. doi: 10.1002/2014JG002874

[B60] Zhou S. Yu B. Zhang Y. Huang Y. Wang G. (2016a). Partitioning evapotranspiration based on the concept of underlying water use efficiency. Water Resour. Res. 52 (2), 1160–1175. doi: 10.1002/2015WR017880

